# (*N*-Alkyloxalamido)-Amino Acid Amides as the Superior Thixotropic Phase Selective Gelators of Petrol and Diesel Fuels

**DOI:** 10.3390/gels9110852

**Published:** 2023-10-27

**Authors:** Nataša Šijaković Vujičić, Janja Makarević, Jasminka Popović, Zoran Štefanić, Mladen Žinić

**Affiliations:** 1 Ruđer Bošković Institute, Division of Organic Chemistry and Biochemistry, Bijenička 54, 10000 Zagreb, Croatia; pmance@irb.hr; 2 Ruđer Bošković Institute, Division of Materials Physics, Laboratory for Synthesis and Crystallography of Functional Materials, Bijenička 54, 10000 Zagreb, Croatia; jasminka.popovic@irb.hr; 3 Ruđer Bošković Institute, Division of Physical Chemistry, Laboratory for Chemical and Biological Crystallography, Bijenička 54, 10000 Zagreb, Croatia; zoran.stefanic@irb.hr; 4 Croatian Academy of Sciences and Arts, Nikole Šubića Zrinskog 11, 10000 Zagreb, Croatia

**Keywords:** organogelator, phase selective gelation, oxalamide, fuel, xerogel structure

## Abstract

(*N*-Alkyloxalamido)-amino acid amides **9**–**12** exhibit excellent gelation capacities toward some lipophilic solvents as well as toward the commercial fuels, petrol and diesel. Gelator **10** exhibits an excellent phase-selective gelation (PSG) ability and also possesses the highest gelation capacity toward petrol and diesel known to date, with minimum gelation concentration (MGC) values (%, *w*/*v*) as low as 0.012 and 0.015, respectively. The self-assembly motif of **10** in petrol and toluene gel fibres is determined from xerogel X-ray powder diffraction (XRPD) data via the simulated annealing procedure (SA) implemented in the EXPO2014 program and refined using the Rietveld method. The elucidated motif is strongly supported by the NMR (NOE and variable temperature) study of **10** toluene-d_8_ gel. It is shown that the triple unidirectional hydrogen bonding between gelator molecules involving oxalamide and carboxamide groups, together with their very low solubility, results in the formation of gel fibres of a very high aspect ratio (d = 10–30 nm, l = 0.6–1.3 μm), resulting in the as-yet unprecedented capacity of gelling commercial fuels. Rheological measurements performed at low concentrations of **10** confirmed the strength of the self-assembled network with the desired thixotropic properties that are advantageous for multiple applications. Instantaneous phase-selective gelation was obtained at room temperature through the addition of the **10** solution to the biphasic mixture of diesel and water in which the carrier solvent was congealed along with the diesel phase. The superior gelling properties and PSG ability of **10** may be used for the development of more efficient marine and surface oil spill recovery and waste water treatment technologies as well as the development of safer fuel storage and transport technologies.

## 1. Introduction

Low-molecular-weight organic gelators (LMWOG) represent interesting soft materials with many possible applications [1,2,3,4,5]. They are used as supports for functional biomaterials, biosensors, and cell scaffolds [6,7,8,9]. There is growing interest in phase-selective gelators (PSG) capable of gelling various oils (or organic phase), including commercial fuels in biphasic oil/water mixtures [10,11]. Since the pioneering work of Bhattacharya and Krishnan-Ghosh [12] on the first phase-selective gelation using an alanine amphiphilic gelator, a perspective for the development of new PSG-based technologies for marine oil spill recovery and the treatment of oil-containing waste water is emerging [13,14,15]. The search for more efficient technologies that lack the disadvantages of the presently used dispersants, sorbents, and solidifiers appears to be of global interest [16,17,18]. Organic gelators suitable for the development of ecologically acceptable PSG technologies are expected to have a very high oil gelation capacity, low production costs, and lack of any toxicity. 

Vibhute and Sureshan published a review on different aspects of the application of PSG gelators in 2020 [19]. Hydrogen bonding, π–π stacking, van der Waal interactions, and other non-covalent attractive forces are responsible for the formation of gels at the molecular level [20,21]. Most of the reported gelators self-assemble via intermolecular hydrogen bonding [22]. 

Phase-selective gelation emerges from the gelator’s selective partitioning to one of the two phases, which is followed by the gelator’s self-assembly. Many examples of phase-selective gelators have been documented, which congeal organic solvents (e.g., benzene, tetrachloromethane, or toluene) from their respective biphasic mixtures with water upon heating and cooling processes [23]. In each of these examples, dissolving the gelator molecules in the organic phase required heating the mixture. This method of phase-selective gelation use cannot be adopted for practical oil spill recovery, since oil spillage happens over a very large water (sea) area where heating or stirring is impossible. Many attempts were made to use PSG as a solution in water-miscible carrier solvents. 

In the case of phase-selective gelation, the water-miscible carrier solvent is dissolved in the aqueous phase and the gelator molecules undergo self-assembly in the oil layer. The formed oil gel can be collected using physical techniques to recover the spilt oil. The solvents used in this application of PSGs are mainly ethanol [12,24], methanol [25,26], THF [27,28], or a mixture of ethanol and ethyl acetate [29]. In these attempts, the organic layer is gelled selectively without affecting the water layer. Unfortunately, water-miscible toxic carrier solvents are harmful to the marine ecosystem. Water-immiscible carrier solvents were studied because they co-congeal with the oil and help avoid the issue of secondary pollution [30]. The gelators were mostly dissolved in toluene or diesel using heat or ultrasound treatment. Afterwards, they were added to a biphasic mixture of water and oil, causing the oil phase to solidify with the carrier solvent and become removable using a spatula. The gelator powder was intended to be directly applied over the spilled oil layer in the fourth procedure. An oil gel network is created when the gelator disperses into the oil layer and self-assembles through non-covalent interactions [31,32]. The wetting approach was used to shorten the gelation time. The phase-selective gelation of different oil layers was achieved using wet powder at room temperature [33,34]. One of the drawbacks of applying gelators (with or without carrier solvents) for oil spill recovery is the difficulty in collecting the gelled oil. Sureshan et al. impregnated the organogelator into the cellulose matrix to create a hybrid sorbent [35]. 

For the past twenty years, Žinić et al. have conducted significant research on the gelation properties of chiral oxalamide derivatives [36]. Strong intermolecular hydrogen-bonding interactions were determined between planar oxalamide units [37]. In-depth research has been carried out on the effects of the hydrogen-bonding unit, the number of those units, the type of chiral centres, and the influence of the lipophilic or hydrophilic terminal groups of the molecules on gelation properties [38].

Very recently, the effect of branched alkyl chain length on the properties of supramolecular organogels from mono-N-alkylated primary oxalamides was reported [39]. The research we propose in this paper on chiral mono-N-alkylated primary oxalamides highlights the importance of the introduction of the chiral units as a prerequisite tool to control the spatial distribution and self-assembly of gelator molecules, providing superior nanostructures capable of gelling various solvents to an extraordinary extent. 

Furthermore, thixotropy is an intriguing property that has garnered a lot of interest in certain organogelling systems [40]. When subjected to external mechanical stress, self-recoverable gels disintegrate in solution and regain their viscoelastic properties after the stress is removed [41]. New insights into the mechanisms of gelation have been gained from the correlations between the thixotropic and structural properties of molecular gels [42], the solvent effect, halogen, and hydrogen bond’s remarkable role on the self-healing supramolecular gels [43], and the impact of H-bonding interactions on the viscoelasticity of the molecular gels [44]. 

Here, we report on the efficient preparation of gelators **9**–**12** that exhibit excellent gelation capacities toward some lipophilic solvents as well as toward the commercial fuels, petrol and diesel. Gelator **10** showing an excellent PSG ability also possesses the highest gelation capacity toward petrol and diesel known to date, with MGC values (%, *w*/*v*) as low as 0.012 and 0.015, respectively [45]. For the first time, the self-assembly motif of **10** in the petrol and toluene gel fibres is determined from the xerogel XRPD data by the simulated annealing procedure (SA) implemented in the EXPO2014 program and the refinement by the Rietveld method. The morphology of the self-assembled gel network was correlated with the gelation capacity and the rheological measurements performed in different solvents. Instantaneous phase selective gelation was obtained at room temperature with the addition of the **10** dichloromethane solution to the biphasic mixture of diesel and water in which the carrier solvent (CH_2_Cl_2_) was congealed along with the diesel phase. Such superior gelling properties as well as the PSG ability may be used for the development of more efficient marine and surface oil spills’ recovery and wastewater treatment technologies as well as the development of safer fuel storage and transport technologies. 

## 2. Results and Discussion

### 2.1. Synthesis and Gelation Properties

**Scheme 1:**
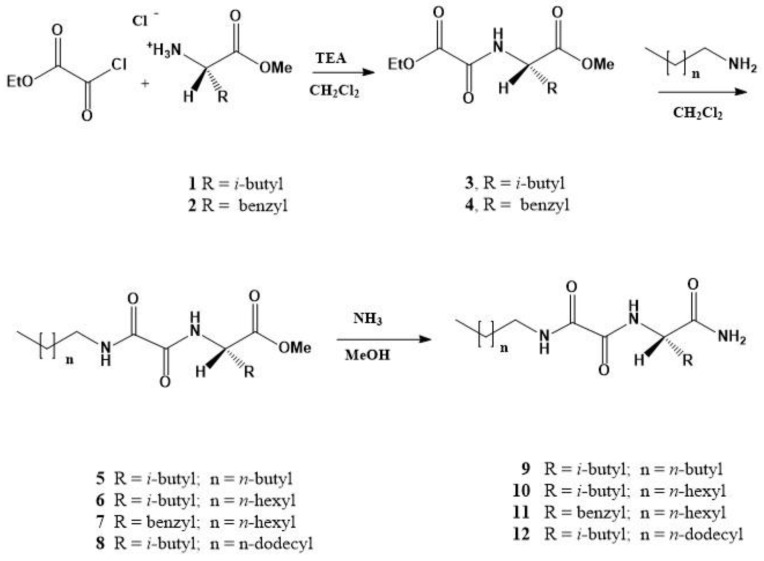
Three step synthesis of (*N*-alkyloxalamido)-amino acid amide gelators **9**–**12**.

*N*-substituted oxalamido-*L*-amino acid amides **9**–**12** are prepared in three steps starting from ethyl oxalylchloride and selected amino acid methyl ester. The resulting ethyl oxalamides **3** or **4** were reacted with the respective alkylamine in CH_2_Cl_2_ giving *N*-alkyloxalamido-*L*-amino acid methyl esters **5**–**8**, which in the reaction with methanolic ammonia gave the amides **9**–**12** in the average overall yields between 80 and 90% (Scheme 1 and Supplementary Materials) [45].

Product **10** of a very high purity crystallises from the reaction mixture as a shining cotton-wool-like material consisting of intertwined tiny crystalline fibres (Figure S1a,b). Formation of such high aspect ratio crystals indicates the predominantly unidirectional packing of the molecules in the solid state.

Examination of gelling properties showed that the compounds **9**–**12** either failed or showed only weak gelation of water and organic solvents of high and medium polarity (EtOH, acetone, acetonitrile, THF and CHCl_3_, Table 1). In contrast, for some aromatic and aliphatic solvents of a low polarity including commercial fuels, **9**, **11**, and **12** exhibited medium to high gelling capacities, except **10** which showed unexpectedly high gelation capacity (MGC % *w*/*v*, g/100 mL: *p*-xylene 0.015; toluene 0.061; decaline 0.009; petrol 0.012, diesel 0.015) [45]. The prepared diesel gel consists of 99.983% of the fuel and 0.017% of **10**; hence, **10** is capable of immobilising the mass of fuel close to 6000 times that of its weight. The examined gelators are 10–100 times more efficient than most of the fuel gelators described in the literature [10].

**Figure 1:**
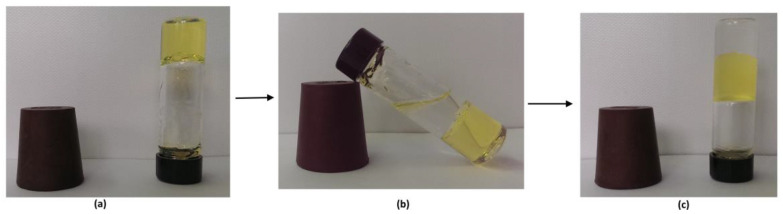
Phase selective diesel gelation (PSG) by gelator **10** in the water/diesel mixture: (**a**) the diesel gel obtained by a heating–cooling procedure of **10** in diesel; (**b**) water added on the top of the diesel gel; (**c**) after heating of the sample presented in (**b**), the diesel gel is dissolved and by shaking with the hot water, the phases were mixed. This was followed by the cooling procedure to room temperature, then the phases were spontaneously separated and ended with the diesel phase gelled on top of the water layer.

In the water/diesel gelling experiments, gelator **10** showed the phase selective gelation of the diesel fuel (Figure 1). The as yet unprecedented gelling capacity of **10** toward petrol and diesel could be of high interest for the development of safer storage and transport technologies; this prospect is also underpinned by the fact that gelled petrol shows 30% decreased vapour pressure (as measured by the Reid method) compared to the fluid petrol sample at 38 °C.

### 2.2. Phase Selective Gelation

Phase selective gelation of the most efficient gelator **10** was investigated in the biphasic mixture of diesel and the water phase. Gelator **10** is capable of congealing the diesel layer from the biphasic mixture with water upon the heating–cooling process (Figure 1). Although this example illustrates the idea of the phase selective gelation, the method of gelation is not applicable for practical oil spill recovery. The oil spillage happens over a large area where heating is practically impossible. Consequently, real oil spills cannot be cleaned up with gelators that need to be heated in order to dissolve in the oil phase. 

**Figure 2:**
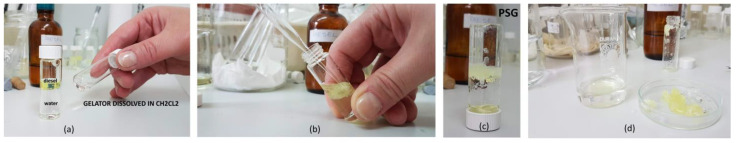
Phase selective gelation process of diesel by gelator **10** (**a**–**c**) and scooped diesel gel (**d**). (**a**) Solution of gelator **10** dissolved in the dichloromethane, (**b**) addition of solution of **10** to the diesel phase followed by instantaneous gelation of the diesel layer at RT, (**c**) formation of diesel gel in the biphasic mixture of diesel and water phase, (**d**) scooped diesel gel and unaffected water phase.

As the next step, we have demonstrated the use of a water-immiscible carrier solvent (CH_2_Cl_2_) for introducing the gelator in the diesel phase. A highly concentrated solution of gelator **10** in CH_2_Cl_2_ solvent is prepared by gentle heating at 35 °C. The formed solution of **10** is stable and applicable at ambient conditions. This solution of PSG is then applied to the diesel layer causing instantaneous gelation of the diesel phase without affecting the water layer. Gelator **10** self-assembles in the diesel layer and congeals the diesel along with the carrier dichloromethane solvent. Thus, this thick, formed gel can be mechanically collected from the water surface (Figure 2). Additionally, the gelled mixture of the carrier solvent and the diesel phase also showed thixotropic properties.

The gelator **10** showed superior critical gelation concentrations (mgcs) and stability of the gels formed. When compared to water-miscible solvents, the employment of water-immiscible carrier solvents that can co-congeal is beneficial. There is no risk from PSG because it is eliminated together with the congealed oil phase. It is advantageous that gelator **10** showed good solubility in the carrier solvent, and the application of a gelator solution in cold conditions provides a practical and feasible method of actual marine oil spills’ recovery. 

The thermoreversibility of low molecular weight organic gelators (LMWOG) is a very important property of stimuli-responsive gels, especially for the oil spill remediation. The recovery of fuel from the gel could be achieved through the distillation process. The gelator can be recovered and reused multiple times. We have demonstrated the recovery of petrol fuel from the **10** petrol gel through the distillation process (Figure S1c). Organogelators acceptable for PSG technologies should have a very high oil gelation capacity, low production costs, and reusability. This represents a promising approach for the treatment of oil spills.

### 2.3. TEM, SEM Microscopy and DSC Study

**Figure 3:**
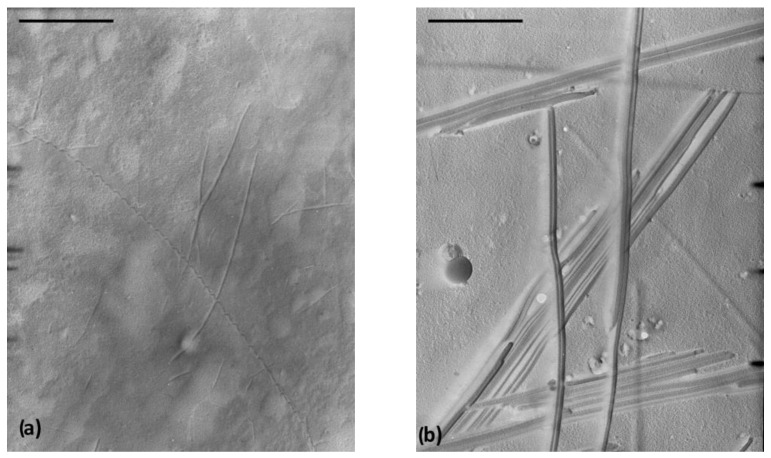
(**a**) TEM images (shadowed with Pd, bar = 1 µm) of **10** diesel gel (the presence of very high aspect ratio fibre, d = 10–30 nm, l > 6 μm) and (**b**) TEM image of **10** toluene gel (fibre bundles, d= 30–100 nm).

Transmission electron microscopy (TEM) investigation of the **10** diesel and toluene gels reveal different gel morphology characterised by the formation of intertwined fibres (diameters 10–30 nm) of a very high aspect ratio in the first gel (Figure 3a) and long fibre bundles (widths, 30–100 nm) in the second gel (Figure 3b). Scanning electron microscopy (SEM) investigation of the petrol and toluene gels leads to similar conclusions relating to the fibre widths (three-fold wider in toluene gel) and a density difference in the self-assembled networks (Figure S2).

The observed morphological difference may explain a lower gelling capacity of **10** toward toluene and higher toward the diesel fuel (Table 1). In gels, a solvent is immobilised by surface tension effects and capillarity, both increasing with descending dimensions of interstices of a self-assembled gel matrix (network) [11,46]. Hence, the gelation capacity of the same gelator toward different solvents could be correlated with fibre dimensions available from electron microscopy studies. Through entanglement, thinner fibres with a high aspect ratio could form a denser matrix containing smaller interstices compared to the system containing thicker fibres (bundles) [47,48]. Consequently, the first system should be capable of immobilising a larger volume of solvent than the second one. Hence, the bundles observed by TEM in the **10** toluene gel could form a less dense network than the tiny fibres and very long fibres of diesel gel so that a larger volume of diesel than toluene could be immobilised by the same amount of gelator **10**. 

All the investigated gels were thermoreversible in nature. Differential scanning calorimetry (DSC) results reveal high thermal stability of petrol and diesel gels showing the gel melting temperatures (*T_m_*, 129.1 and 154.3 °C at c = 0.025 M, respectively) and the gel melting enthalpies (Δ*H_m_*, 67.9 and 47.2 kJ/mol, respectively; Table S2). The measured gel melting enthalpy change in the **10** petrol gel exhibits a higher Δ*H_m_* value compared with the gels in diesel and decaline. The solubility and the strength of hydrogen bonding as well as lipophilic interactions that stabilise the gel aggregates in solvents of a different polarity are among the variables that affect the gel melting enthalpies. 

### 2.4. Crystal Structure and XRPD Studies

**Figure 4:**
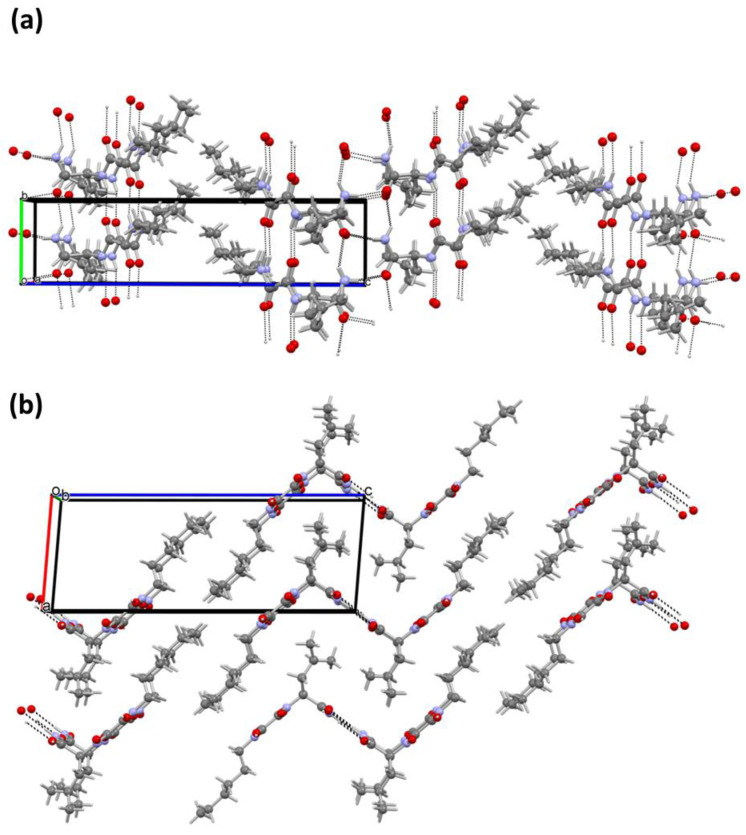
Crystal packing of **10**. (**a**) Two sets of hydrogen bonds connect the molecules in the direction of b crystallographic axis: one forms the ladder-like pattern between the central oxalamide bridges and the other connects these ladders sideways; (**b**) view along the b axis reveals that the crystal packing is comprised of central hydrophilic hydrogen-bonded chains surrounded by hydrophobic domains. Crystallographic axis a is shown with the red line, while the b- and c-axis are shown in green and blue, respectively.

The molecular structure of **10** has been determined by single-crystal X-ray diffraction analysis (Figure S3). The compound crystallised from methanol in the monoclinic space group *P* 2_1_ with one molecule in the asymmetric unit. Crystallographic data, data collection, and structure refinement details are provided in Table S2. Molecules are connected by two N-H···O hydrogen bonds between central oxalamide bridges (Table S3; hydrogen bonds a and b), a pattern characteristic of the oxalamide class of compounds [38]. These two hydrogen bonds exist between the molecules connected by pure translation in the direction of the crystallographic b axis (Figure 4a). In this way, the ladder-like pattern is formed in the b direction. The molecule of **10** is asymmetric with respect to the central C1–C2 bond. The leucine moiety on one side makes another pair of N-H···O hydrogen bonds (e and f in Table S3, Supplementary Materials) with the oxygen atoms from two different neighbouring molecules. In this way, another chain of the hydrogen bonds is formed along the 2_1_ crystallographic screw axis (Figure 4a). On the other side of the oxalamide bridge, the molecule has no hydrogen bonding capability and therefore makes a long hydrophobic region with similar alkyl chains from the neighbouring molecules. This part is quite disordered as can be seen from exceptionally high thermal ellipsoids from terminal C14 atoms (Figure 4b). Overall, the crystal packing of **10** can be viewed as chains of hydrogen bonded molecules along the crystallographic b axis. The central hydrogen bonded part forms a zig-zag shape and around it hydrophobic parts are placed consisting of leucine and C9–C14 alkyl chains.

**Figure 5:**
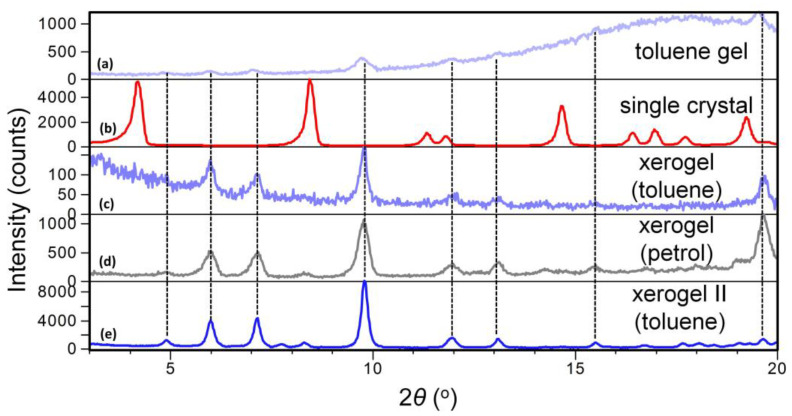
XRPD patterns of compound **10** prepared form: (a) toluene gel (c = 4.7 × 10^−3^ M); (b) single crystal obtained by crystallisation from methanol; (c) xerogel prepared from toluene gel (c = 4.7 × 10^−3^ M); (d) xerogel prepared from petrol gel (c = 3.5 × 10^−2^ M); (e) xerogel II prepared from more concentrated toluene gel (c = 7.0 × 10^−2^ M).

To find out if the same or a different self-assembly motif of **10** exists in the single crystal, petrol, and toluene gels, the X-ray powder diffraction data (XRPD) of the single crystal (methanol) and the petrol and toluene xerogels are compared. Figure 5 shows XRPD patterns of: (a) **10** toluene gel, (b) **10** single crystal (methanol), (c) xerogel (from **10** toluene gel) and (d) xerogel (from **10** petrol gel) and (e) xerogel II (from higher concentration **10** toluene gel). Although the XRDP pattern of toluene gel (Figure 5a) shows a typical amorphous halo, from the position of the Bragg reflections it can be concluded that the molecular arrangement in the gel phase closely corresponds to those in toluene (Figure 5c) and petrol (Figure 5d) xerogels while it is clearly different to that in the single crystal determined in methanol (Figure 5b). Also, the high similarity between XRPD patterns of the petrol and toluene xerogels points out that no significant difference in the molecular assembly should be expected with respect to the solvent. 

**Figure 6:**
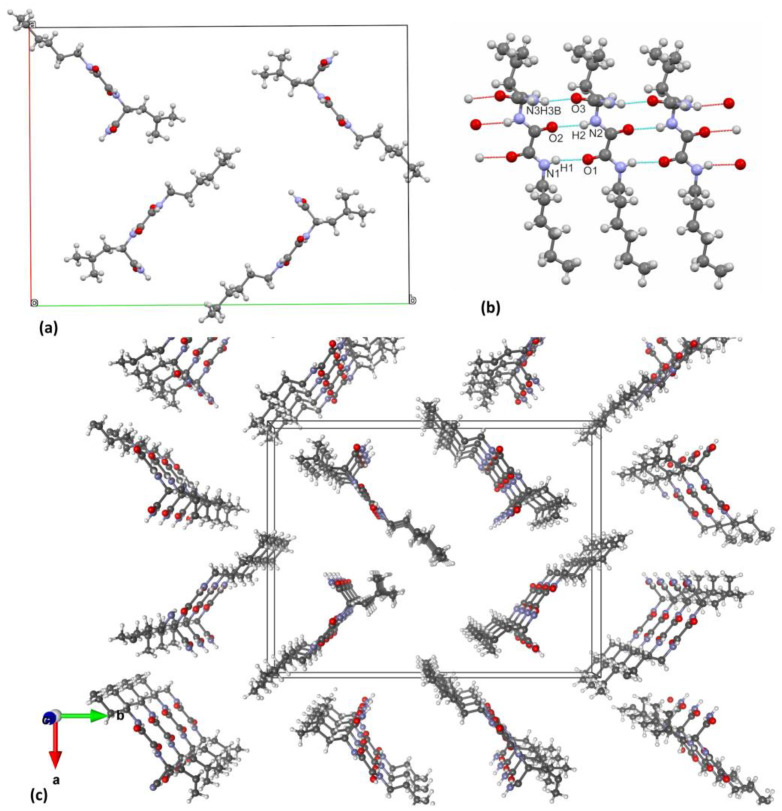
Structure of **10** toluene xerogel. (**a**) Arrangement of the molecules within the unit cell viewed in the *ab*-plane, (**b**) intermolecular hydrogen bonds running parallel to the *c*-direction and (**c**) perspective view at 1D chains formed via H-bonded molecules in the *c*-direction. Crystallographic axis *a* is shown with the red line, while the *b*- and *c*-axis are shown in green and blue, respectively.

The structure of the xerogel has been solved by a simulated annealing procedure (SA) implemented in the EXPO2014 program and refined by the Rietveld method (see Supplementary Materials for details and Figure S4). To the best of our knowledge, there are very few examples of such a structure determination presented in the literature until now for the xerogels of the low molecular weight organogelators [49,50,51]. The toluene xerogel crystallises in orthorhombic system, in non-centrosymmetric, space group P 2_1_ 2_1_ 2_1_ with the unit cell parameters a = 22.819(1) Å, b =29.498(1) Å and c = 5.095(1) Å. The unit cell consists of four molecules as shown in Figure 6a. The molecules are interconnected by three N-H…O type hydrogen bonds (oxalamide fragments and amide groups) forming 1D chains parallel to the *c*-direction as displayed in Figure 6b,c (Table S4). Hydrogen bonds between the amide groups (N3–H3B…O3) are slightly shorter than the H-bonds between the oxalamide groups (N2–H2…O2 and N1–H1…O1). Interestingly, only one amide hydrogen (H3B) participates in intermolecular hydrogen bonding with the carbonyl oxygen (O3) from the neighbouring molecule (Table S5).

In contrast to the **10** single crystal structure (Figure 4), where oxalamide and amide intermolecular hydrogen bonding occurs in two perpendicular planes inducing self-assembly in two directions, in the xerogel structure (Figure 6) the molecules of **10** are connected by three in the plane oxalamide and amide hydrogen bonds. Since hydrogen bonding is highly favoured in lipophilic solvents such as petrol or toluene, a fast and predominantly unidirectional self-assembly of **10** is expected providing the fibres with a high aspect ratio. By entanglement, such fibres are capable of forming the dense gel network and immobilising large volumes of solvent. However, the gelation capacity of **10** is close to five times higher toward petrol than toward toluene in an agreement with the observation of the thinner fibres in the first and thicker fibre bundle in the second gel (Figure 3a,b and Figure S2). Since a very similar molecular arrangement of **10** in petrol and toluene xerogels is found (XRPD, Figure 5) the apparent morphological difference between the petrol and toluene gels could be explained by the solvent effects [52,53,54,55,56,57]. In toluene, the less favourable solvent–aggregate interactions may induce fibre bundling in order to diminish the solvation surface which ultimately results in the formation of a less dense gel network and immobilisation of a lower volume of the solvent.

### 2.5. NMR and FTIR Study 

NMR is frequently used in studies of the gels providing gel phase information on the gelator organisation in gel fibres [58]. It was shown that smaller aggregates dissolved in the entrapped solvent can be observed by NMR spectroscopy, and these are organised similarly to those in the gel fibres [39,52]. As described above, **10** single crystal and XRPD xerogel studies have shown that the molecular arrangement of **10** in the toluene and petrol gel phases (i.e., its self-assembly motif in gel fibres) closely corresponds to those in toluene (Figure 5c) and petrol (Figure 5d) xerogels while it is clearly different to that in the **10** single crystal (Figure 5b). However, a solid–solid morphological change in the gel fibres during its transformation from the gel to xerogel can be induced either by the solvent removal or by nucleation initiated by the small amount of gelator that may have been present in the bulk liquid in a gelled state. Therefore, there is no certainty that the molecular packing in the fibre of a xerogel truly represents that in the gelled state [59]. In order to acquire the structural information directly from the gel phase as support to the organisation of **10** in toluene and petrol xerogels (Figure 5), NOESY and the variable temperature NMR experiments were executed. 

In the NOESY spectra of **10** toluene-d_8_ gel taken at 65 °C, the NOE effects assigned to the intramolecular interactions (Figure S5, intramolecular H/H distance below 5 Å) could be observed in the addition to the interaction between α-methylene protons of *N*-hexyl group (δ 3.0 ppm) and the signal of Leu and hexyl methyls appearing at δ 0.8 ppm which cannot be of the intramolecular origin since the distance between these groups exceeds 5 Å. Hence, this NOE effect could be explained by the intermolecular interaction between the Leu methyl hydrogens and the *N*-hexyl α-methylene group which stands in the agreement with the packing of **10** found in the toluene and petrol xerogels (Figure 6c, H/H distance 4.7 Å) where these groups are in the close proximity. 

**Figure 7:**
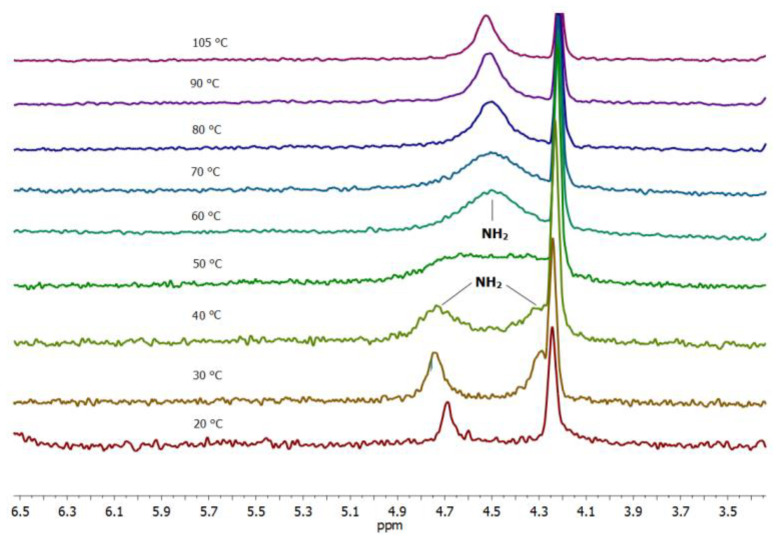
Temperature dependant NMR spectra of **10** toluene-d_8_ gel (carboxamide region; 25–100 °C interval) showing two different amide NH signals (δ 4.78 and 4.35 ppm) in the gel state at 20–40 °C and a single broadened signal in the sol at 70–105 °C.

Another striking feature of the **10** xerogel crystal structure is the participation of only one amide hydrogen in the intermolecular hydrogen bonding (Figure 6b). To find out if the same feature holds for the gel phase the variable temperature experiments were performed with the **10** toluene-d_8_ gel. Figure 7 shows the spectral changes appearing in the carboxamide region of the proton NMR spectra taken in the 20−105 °C temperature intervals. In the gel state at 20–40 °C, two well-separated signals at δ 4.69 and 4.25 ppm of the two non-equivalent amide NHs can be observed.

An increase in the sample temperature results in a gel-to-sol transition between 40 and 50 °C (*T_g_* 45 °C) followed by the appearance of a single broad signal for two amide NHs. The non-equivalency of the two amide NHs in the gel phase could be explained by the participation of the one amide NH (δ 4.78 ppm) in the intermolecular hydrogen bonding in the gel aggregates and the absence of such interaction for the second amide NH appearing at δ 4.35 ppm. The temperature increase leads to dissociation of the gel aggregates and breakage of hydrogen bonds which is reflected in the appearance of a single broadened amide NH_2_ signal. The chemical shift changes in the oxalamide LeuNH (δ 7.84) and hexyl-NH (δ 7.22.ppm) proton region during the temperature variation are in accord with their participation in the intermolecular hydrogen bonding in the gel aggregates (Figure S6). Through the temperature increase, both the LeuNH and the hexyl-NH oxalamide protons are shifted upfield (Figure S6) in accordance with dissociation of the gel aggregates and breakage of the intermolecular hydrogen bonds. The hexyl-NH oxalamide protons are positioned in ^1^H-NMR spectra below the toluene-d_8_ signals. The hexyl-NH oxalamide protons were seen only for high concentrations of **10** toluene-d_8_ gels at temperatures higher than 70 °C. In conclusion, the structural information gained from the NOESY and variable temperature experiments on **10** toluene-d_8_ gel is in agreement with the organisation of **10** found in the crystal structure of the xerogel. 

Additionally, FTIR measurements of the **10** crystal and diesel gel samples were performed in the pastille at room temperature. XRPD diffraction and NMR experiments provided evidence of the two non-equivalent amide NHs, one involved in the intermolecular hydrogen bonding in the gel aggregates and the second without participation in the hydrogen bonding. The FTIR spectra provided the existence of an additional signal at 3425 cm^−1^ in the NH region for **10** diesel gel compared to the crystal spectra obtained from methanol (Figure S7). The position of the signal corresponds to the free NH hydrogen of the primary amide of **10** in the gel state. NMR and FTIR measurements examined in toluene and diesel gels confirmed the premise of the molecular organisation in the petrol gel elucidated from the xerogel XRPD data. 

### 2.6. Oscillatory Rheology of the Gels

#### 2.6.1. Amplitude Sweep

The self-assembled fibre network’s characteristics, including its length, thickness, the number and the type of non-covalent interactions, and spatial distribution, determine the viscoelastic properties of the gels. The viscosity decreased as the shear rate increased in all of the gels under examination, exhibiting a non-Newtonian shear-thinning behaviour. The elastic component of the viscoelastic behaviour, which characterises the sample’s solid state, is represented by the storage modulus G′ (Pa). In gels, with a small applied shear, the elastic modulus (G′) predominates over the loss modulus (G″), achieving a plateau in the linear viscoelastic region (LVR). The range in which the applied stress has no effect on the gel’s structure is known as the linear viscoelastic region. Up to the yield strain, the material’s rheological characteristics are unaffected by a strain; after that point, the viscoelastic behaviour becomes nonlinear. 

**Figure 8:**
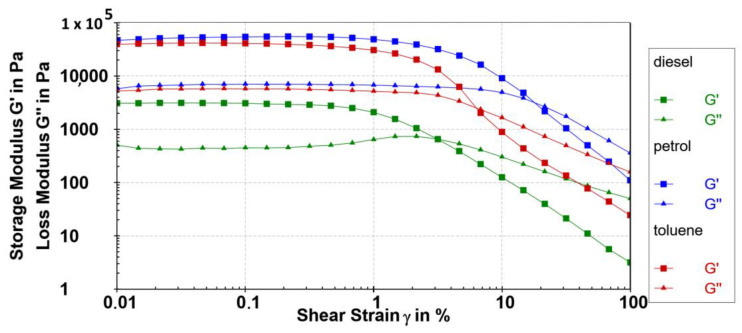
Amplitude sweeps tests (G′ (■) and G″ (▲) values) of the 0.2 wt% **10** petrol gel (blue), toluene gel (red), and diesel gel (green) at 10 °C.

The mechanical properties of the **10** diesel, petrol, and toluene gels were described using the oscillatory rheology. Strain sweep tests were performed in the range of γ = 0.01 to 100% at ω = 10 rads^−1^ of gelator **10** at 0.2 wt% at 10 °C. The G′ and G′′ values remained approximately independent of the applied strain up to 0.3%. Figure 8 shows strain sweeps for the corresponding 0.2 wt% gelator **10** in petrol, toluene, and diesel gels, respectively. The high values of the elastic portion or storage modulus (10^4^ Pa) measured by the amplitude sweep tests resemble the strength of the examined gels at only 0.2 wt%. The storage modulus (G′) value (determined at LVR, 10 rads^−1^; Figure 8) of 0.2 wt% **10** petrol gel (G′ = 5.5 × 10^4^ Pa) is higher than that of the toluene gel (4.2 × 10^4^ Pa) and more than one order of magnitude higher compared to the diesel gel (3.1 × 10^3^ Pa) (Table 2).

The yield stress of the **10** petrol gel is 43 times higher compared to the **10** diesel gel examined at the same concentration. Compared to the moderate strength of the diesel gel, the **10** petrol gel showed the highest storage moduli values, a broader linear response region, as well as a much higher yield point value. The largest yield stress and yield strain value of 0.2 wt% **10** petrol gel were even 828.5 Pa and 3.24%, respectively. Very high values were also determined for toluene gel (393.5 Pa, 1.49%) and were moderate for diesel gel (18.87 Pa, 0.78%). 

The relative elasticity of viscoelastic materials is defined by the rheologically derived quantity tan(δ) = G″/G′. The gels formed in all investigated solvents showed low tan(δ) values in a range from 0.12 in petrol to 0.14 in diesel gel, which is indicative of a very high relative elasticity in all examined solvents. The gels with a value of tan(δ) = 0.1 belong to stiff gels. The investigated gels in petrol, toluene, and diesel (loss factor or tan(δ) values 0.12; 0.13 and 0.14, respectively) were stiff gels even at the examined concentration of 0.2 wt% of the gelator **10**. 

The oscillatory sweep results showed the powerful influence of the different chemical composition of various fuels and solvents on the solubility of the compounds and the self-assembling potential leading to various gelation potencies and consequently the different viscoelastic properties of the formed gels. Lower elastic modulus and yield point values determined in the diesel gel compared to the petrol and toluene gels are evidence of the influence of the solubility of the gelator and the chemical composition of the solvents on the viscoelastic properties of the new gel materials. 

#### 2.6.2. Frequency Sweep

**Figure 9:**
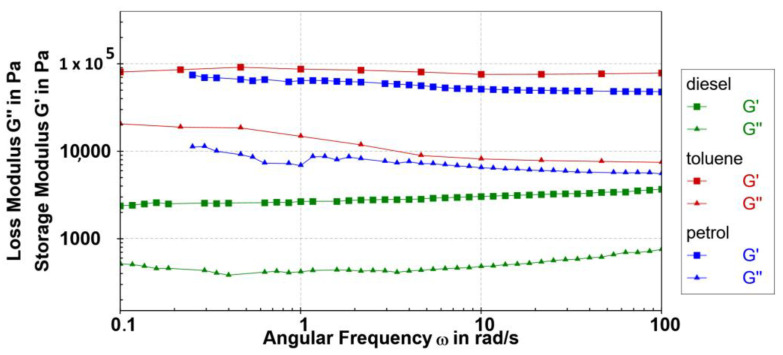
Frequency sweep tests (G′ (■) and G″ (▲) values) of 0.2 wt% **10** petrol gel (blue), toluene gel (red) and diesel gel (green) were performed at 0.1% strain at 5 °C.

The frequency sweep investigations conducted on the studied gels (ω = 0.1–100 rad/s at 0.1% strain) show that, within the linear viscoelastic zones (LVRs), the values of the storage modulus (G′) and loss modulus (G″) are mostly independent of the applied frequency (Figure 9). The investigated samples are gels with approximately maintained relative elasticity (tan δ~0.13) throughout the entire examined range of frequencies. Despite the condition of the hood presence, a small deviation of elastic moduli was observed at lower frequencies due to evaporation of petrol and toluene solvents because of a prolonged period for the measurements. Additionally, a slight tendency of wide fibres and crystal formation at higher concentrations in toluene and petrol gels was observed over time by visual observation in the test tubes.

The gels formed in petrol and toluene showed maintained relative elasticity at higher frequencies compared to diesel gel with a slight change in elasticity at higher frequencies due to the existence of a softer gel network. Nevertheless, the **10** diesel gel showed an extraordinary stability over years. 

#### 2.6.3. Thixotropic Properties

One of the most crucial rheological characteristics to take into account when creating gels is the thixotropic structure recovery since it significantly affects the gel’s practical use. The most appropriate technique for structural recovery tests is the three-interval thixotropy test (3ITT), a standard procedure that enables tracking of a material response resulting from the stepwise changes in the shear strain. 

**Figure 10:**
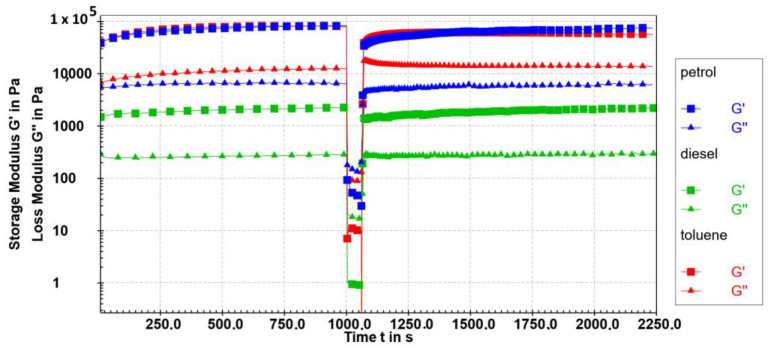
The 3-interval thixotropy test (G′ (■) and G″ (▲) values) of 0.2 wt% of **10** petrol gel (blue), toluene gel (red), and diesel gel (green) at 10 °C as a function of time and strain application. Linear viscoelastic region conditions (LVR): γ = 0.1%, ω = 10 rad/s; destructive region (DR): strain = 100%, frequency = 10 rad/s; recovery region, linear viscoelastic region (LVR): strain = 0.1%, frequency = 10 rad/s.

The possibility that **10** gels in diesel, petrol, and toluene are thixotropic was explored. In the thixotropic studies, baseline values for G′ and G″ were determined by performing the rheological measurements on the fresh gels at 10 °C under initial conditions where they were in their linear viscoelastic regimes (a strain of 0.1% and an angular frequency of 10 rad/s) for 1000 s. The evolution of G′ and G″ for the 0.2 wt% **10** gels in toluene, diesel, and petrol is depicted in Figure 10 following the application of 100% strain, which causes the gel to lose its viscoelasticity. Following the end of the destructive strain, the initial conditions were once again applied to track the gels’ ability to regain their viscoelastic properties. Approximately 50–63% of the original G′ values were recovered in less than 30 s. The fastest recovery after 30 s was observed in diesel gel (63%), then toluene gel (57%), and then in petrol gel (48%) (Table 3). Then, after 5 min, the recovery of viscoelasticity was 77% for toluene gel, 76% for diesel gel, and 71% for petrol gel. Nevertheless, for all investigated samples, 50% of the original G′ values were recovered in 10−30 s. 

The visual appearance of the diesel gel did not change over a prolonged period of time, contrary to toluene and petrol gels which, depending on the concentration, eventually tended to form a wider fibre network over time, and even a multi-domain crystalline network homogenously distributed through the gel samples. 

Independent of the recovery ratio, the relative elasticity is maintained in the diesel and petrol gel after the cessation of the destructive strain; however, in the toluene gel a higher value of the loss factor 0.22 was determined in the recovery interval, compared to 0.15 in the first non-destructive interval. Nevertheless, comparing the observations noticed in the oscillatory measurements with the gelation efficiencies determined by the test tube inversion method, one could expect a more efficient recovery in the systems of higher gelation potency (as in the diesel and petrol gels) and a uniform network constituted of tiny fibres (diesel and petrol) contrary to the wider fibre bundles observed in toluene gel. The thixotropic properties of the low molecular weight gelators are still a rare phenomena not yet investigated in detail [44]. The potency of the thixotropic organogelators presented in this paper is enormous. The gelators showed potential to be applied as the phase selective gelators; moreover, the fact of being thixotropic in nature improves their practical feasibility even more significantly.

## 3. Conclusions

(*N*-Alkyloxalamido)-amino acid amide gelators **9**–**12** exhibit excellent gelation capacities toward some lipophilic solvents as well as toward the commercial fuels, petrol and diesel (Table 1). Gelator **10** shows an excellent phase selective gelation (PSG) ability and also possesses the highest gelation capacity toward petrol and diesel known to date, with MGC values (%, *w*/*v*) as low as 0.012 and 0.015, respectively. The single crystal and XRPD study of the **10** toluene gel and petrol and toluene xerogels reveal that the organisation of **10** is practically identical in the toluene gel and the petrol and toluene xerogels are clearly different from the packing in **10** single crystal. For the first time, the self-assembly motif of **10** in the petrol and toluene gel fibres is determined indirectly from its xerogel crystal structure determined from the xerogel XRPD data by the simulated annealing procedure (SA) implemented in the EXPO2014 program and refinement by the Rietveld method. The elucidated motif is strongly supported by the NMR (NOE and variable temperature) study of **10** toluene-d_8_ gel. The results obtained reveal that the triple unidirectional hydrogen bonding between gelator molecules involving oxalamide and carboxamide groups, together with its very low solubility, results in the formation of gel fibres of a very high aspect ratio (d = 10–30 nm, l = 0.6–1.3 μm) both yielding the yet unprecedented capacity of gelling commercial fuels. The strength of the examined gels, the high yield point values, low relative elasticity, the stability determined in frequency measurements and the thixotropic behaviour represent powerful viscoelastic gel materials. Such superior gelling properties as well as the PSG ability may be used for the development of more efficient marine and surface oil spill recovery and waste water treatment technologies as well as the development of safer fuel storage and transport technologies. Instantaneous phase selective gelation was obtained at room temperature with the addition of the solution of **10** to the biphasic mixture of diesel and water in which the carrier solvent was congealed along with the diesel phase. It is advantageous that the gelator **10** showed good solubility in the carrier solvent, and the application of a gelator solution in cold conditions merits the practical and feasible method of the actual marine oil spill recovery. It should be noted that further, straightforward synthetic modifications of this type of gelator by using various amino acids and alkyl chains may result in the preparation of even more efficient gelators for commercial fuels and open the way to their technological application. 

## 4. Materials and Methods

The synthesis of the compounds **9**–**12** were prepared according to the procedure described in the patent of Žinić et al. [45]. The synthetic procedure is described in the Supplementary Materials. 

### 4.1. Determination of Gelling Properties

Every gelation experiment was carried out in test tubes with a diameter of 12 mm. A total of 500 µL of the solvent was introduced to a test tube containing the compound being evaluated. Once the solvent was added, the mixture was heated gradually until the material dissolved. It was then allowed to spontaneously cool to room temperature, and the development of a gel was verified by inverting the test tube. The procedure was repeated until the formation of a loose gel or dissolution was observed. The gelation properties of prepared compounds were tested against different solvents and the results are collected in Table 1. The amount of solvent that each gelator can immobilise with 10 mg of gelator is represented in mL as the gelation efficiency towards the designated solvent. All of the prepared gels showed thermoreversible gel-to-sol transitions. 

### 4.2. TEM Investigation

A Zeiss EM 10A transmission electron microscope (Zeiss, Jena, Germany) running at 60 kV was used to examine the specimens. A portion of the gel was placed on a copper grid for electron microscopy, and after 20 s, it was removed, leaving some patches of the gel on the grid. Morphologies of the self-assembled fibres of the gelators were negatively stained by dipotassium polytungstate and Pd shadowing. 

### 4.3. SEM Microscopy

JEOL JSM-5800 scanning electron microscope (Jeol Ltd., Tokyo, Japan) was used for taking SEM images. The frozen gel specimen (liquid nitrogen) was evaporated using a vacuum pump for 24 h. The dry sample was shielded with gold (10 Å). The accelerating voltage of SEM was 20 kV. 

### 4.4. NMR Spectroscopy

With Bruker AV-300 and AV-600 spectrometers (Bruker Biospin GmbH, Rheinstetten, Germany) operating at 300 and 600 MHz for the 1H nucleus and 75 and 150 MHz for the 13C nucleus, respectively, one- and two-dimensional homo- and heteronuclear 1H and 13C NMR spectra were acquired. Chemical shifts (δ) were reported in ppm, coupling constants (J) in Hz. The spin multiplicities are as follows: s (singlet), d (doublet), t (triplet), q (quadruplet), p (pentet), and m (multiplet). 

### 4.5. Differential Scanning Calorimetry

Using a Perkin-Elmer DSC 7 (Perkin Elmer Ltd., Waltham, MA, USA) differential scanning calorimetry (DSC) was performed. The gels were measured by filling a 60 µL stainless steel sample cup with weighed quantities of both the gelator and solvent, then sealing the cup immediately. The sample cup was placed in the DSC apparatus together with an empty sample cup as a reference. At a scan rate of 5 °C min^−1^, the heating and cooling scans were obtained. Repeated heating and cooling of the samples were reproducible. 

### 4.6. FTIR Measurements

Wave numbers (*ν*) are given in cm^−1^ for FTIR spectra obtained in KBr pellets using an ABB Bomen MB 102 FTIR spectrometer (Quebec City, QC, Canada). Additionally, the FTIR investigations on the gels were conducted in the sealed heatable cells for liquids (path length 0.05 mm, CaF_2_ windows). 

### 4.7. Single Crystal Analysis

Crystals of **10** suitable for the single-crystal X-ray analysis were grown from methanol by slow evaporation at room temperature. At room temperature, the data collection was performed on Xcalibur Nova X-ray diffractometer CCD system (Agilent Technologies UK Ltd., Oxford, UK) with multilayer optics and Cu Kα radiation (λ = 1.5412 Å). Data reduction was performed using CrySalisPro software system, Version 71.37.35. [60] and the WINGX [61] program package. The structures were solved by direct methods using the SHELXS package and refined by SHELXL-2013 [62]. Table 1 provides information on the determination and refinement of the crystal structure. MERCURY generated plots of the molecule and crystal packings [63]. Atomic scattering factors were included in SHELXL-2013. All hydrogen atom coordinates, with the exception of those engaged in hydrogen bonds, which were found in electron density, were determined geometrically and refined using the riding model of the same package. Crystallographic data for the structure of **10** are deposited in the Cambridge Structural Database under the CCDC 1523449.

### 4.8. XRPD Studies

X-ray powder diffraction data were collected on a Philips MPD 1820 (Almelo, Netherlands) with CuK_alpha_ radiation in the range 2.7–20.0° 2θ, with a step of 0.2° and a fixed counting time of 1 s per step. Additionally, the xerogel sample was ground in a mortar for 3 h (in order to avoid possible preferred orientation) and measured in the range 2θ 2.7–40.0° with a counting time of 10 s per step with the purpose of space group determination and structure solution. 

### 4.9. Structure Solution from XRPD Data

The structure of xerogel was solved by a simulated annealing procedure (SA) implemented in the program EXPO2014 [64] and refined by the Rietveld method. Program N-TREOR09 was used for the indexing procedure [65]. The proposed cell with the highest figure of merit was chosen; figure-of-merit M_20_ = 21 [66] and figure-of-merit FOM_new_ = 2.0 [67]. The statistics of the normalised *z* intensities (extracted by the Le Bail method [67]), were utilised to calculate the probability extinction symbol compatible with the crystal system suggested by N-TREOR09. Based on this procedure, the extinction group P 2_1_ 2_1_ 2_1_, characterised with figure-of-merit FM = 0.798, was chosen for further structure solution steps. The structure was solved in the process of generating a random sequence of trial structures starting from the model obtained from the single crystal data. The model was allowed to translate (along the *x*, *y* and *z* axis) and rotate within the orthorhombic cell until a satisfactory agreement between the calculated and the observed pattern was found. Bond distances and angles were kept fixed while only the torsion angles were varied during the SA procedure. Crystallographic data for the structure of **10** (determined from xerogel) are deposited in the Cambridge Structural Database under the CCDC 1524532.

### 4.10. Rheological Investigation

Oscillatory rheology was used to characterise the mechanical characteristics of the gels. Using a steel plate-plate geometry (PP25, rubbed surface) and a mechanical rheometer (Anton Paar MCR 302; Ostfildern, Germany) with a true-gap system, the storage (G′) and loss (G″) moduli of the gels were obtained. RheoCompass 1.21 software was utilised to collect the data. A Peltier temperature control located at the geometry’s base and a Peltier-controlled hood (H-PTD 200) were used to regulate the sample’s temperature. A 1 mm thick slice of the gel sample was put on the rheometer’s base plate, and the plate was adjusted using the software’s true-gap feature. The yield stress of the gels was assessed by applying a strain (*γ*) sweep between 0.01% and 100% after 10 min at 10 °C. A gel’s rheological characteristics are strain-independent up to the yield strain, after which their behaviour becomes nonlinear. The most acceptable method for structural recovery tests is the three-interval thixotropy test, a standard test that tracks the material response resulting from incremental changes in the shear strain. In the thixotropic studies, baseline values for G′ and G″ were established by performing the rheological measurements on gels for 1000 s at 10 °C when they were in their linear viscoelastic regimes (a strain of 0.1% and an angular frequency of 10 rad/s). The viscoelastic recovery after the cessation of a destructive strain was assessed in gel investigations. In order to examine the time-dependent deformation behaviour of the gels, frequency sweeps (0.01–100 rad/s) were subsequently carried out at 5 °C at a strain value within LVR. 

## 5. Patents

Žinić, M.; Makarević, J. Amphiphilic oxalamide organogelators designed for gelation of organic solvents, water and hydrocarbon commercial fuels. US Patent No. 8,637,705 B2, 28 January 2014; European Patent EP2188246B1, 17 June 2015; Eurasian Patent No. 021 031, 31 March 2015.

## Figures and Tables

**Table 1:** Maximum volume of solvent or solvent mixture (in mL) gelled by 10 mg of gelator **9**–**12**.

		V/mL		
	9	10	11	12
petrol	45.80	79.20	7.00	10.30
diesel	36.10	67.20	10.0	42.40
water	6.85	NG	NS	NG
DMSO	NG	NG	NG	NG
DMSO/water	1.5 + 0.75	2.15 + 1.65	7.50 + 20.50	17.10 + 20.70
DMF/water	0.6 + 0.5	1.15 + 2.55	3.45 + 3.45	10.35 + 13.30
EtOH	0.25	NG	NG	1.05
THF	0.1	NG	0.50	0.15
EtOAc	0.2	NG	1.00	NG
acetone	NG	NG	NG	NG
CH_2_Cl_2_	1.05	750	NS	750
CH_2_Cl_2_/DMSO				0.40 + 0.04
CH_3_CN	NG	NG	NG	1.45
toluene	24.95	16.20	4.00	15.20
*p*-xylene	33.60	68.00	7.00	5.25
decaline	37.40	106.25	26.00	24.10
tetraline	0.5	0.15	0.50	0.30

NG = no gelation.

**Table 2:** Results of the amplitude sweep tests of the 0.2 wt% of **10** petrol, toluene, and diesel gels examined at 10 °C.

				Yield Point/Pa	
Gelator 10	G′/Pa	G′′/Pa	tanδ	τ/Pa	γ/%
petrol	54,996.0	7002.1	0.12	828.5	3.24
toluene	41,623.3	5738.1	0.13	393.5	1.49
diesel	3105.5	443.3	0.14	18.87	0.78

**Table 3:** Self-healing properties of the 0.2 wt% of **10** petrol, diesel, and toluene gels were determined in the 3-interval thixotropy test at 10 °C.

3ITT Test		
Sample 10	Recovery (t = 30 s)/%	Recovery (t = 300 s)/%
petrol	50.1	71.2
toluene	56.7	77.1
diesel	62.6	76.2

## Data Availability

The data presented in this study are available in the Supplementary Materials.

## Supplementary Materials

The following supporting information can be downloaded at: https://www.mdpi.com/article/10.3390/gels9110852/s1, Figure S1. (a,b) compound **10** isolated in high purity from methanol solution as the shining cotton-wool like material; (c) recovery of petrol by distillation of **10** petrol gel. Figure S2. SEM images of **10** (a) toluene gel and (b) petrol gel; Table S1. Δ*H_m_*, *T_m_* and *T_c_* values obtained from DSC measurements of petrol, diesel and decaline gel of compound **10** in the heating and cooling cycle; Table S2. crystallographic data, structure solution, and refinement for **10** (methanol); Table S3. hydrogen bonds for **10** [Å and °]; Figure S3. molecular structure of **10** with the atomic numbering. Displacement ellipsoids are drawn at the 50% probability level. It is visible that the alkyl chain C9–C14 is highly disordered; Figure S4. Rietveld refinements on compound **10** in the form of xerogel from toluene. Experimental data are shown as red dots, a calculated profile is shown in blue while the difference curve is provided below. Green vertical marks represent diffraction line positions of compound **10** in the form of xerogel; Table S4. crystallographic data, structure solution, and refinement for **10** (toluene xerogel); Table S5. hydrogen bonds for **10** toluene xerogel [Å and °]; Figure S5. NOSY spectrum of **10** toluene-d_8_ gel taken at 65 °C with indicated intramolecular (black lines) and intermolecular (red lines) N-hexyl α-methylene (δ 3.0 ppm)/Leu methyl (δ 0.8 ppm) interactions; Figure S6. downfield shifts of the oxalamide LeuNH protons by the temperature variation from 20 to 105 °C in the NMR spectra of **10** toluene–d_8_ gel. The slope of the red line indicates the extent of temperature−induced downfield shifts for LeuNH protons. The hexylNH protons are positioned under signals of toluene-d_8_; Figure S7. FTIR measurements of **10** crystal (black), diesel gel (red) and diesel solvent (green) at 25 °C.
