# Supplementary material for: (N-Alkyloxalamido)-Amino Acid Amides as the Superior Thixotropic Phase Selective Gelators of Petrol and Diesel Fuels
Source: Gels. 2023 Oct 27;9(11):852. doi: 10.3390/gels9110852 (PMC10670479; doi:10.3390/gels9110852)
Supplement: Supplementary file 1 [file gels-09-00852-s001.zip › gels-2670082-supplementary.pdf]

Supporting Material

(*N*-Alkyloxalamido)-Amino Acid Amides as the Superior Thixotropic Phase Selective Gelators of Petrol and Diesel Fuels

Nataša Šijaković Vujičić [1,*], Janja Makarević [1], Jasminka Popović [2], Zoran Štefanić [3] and Mladen Žinić [4,*]

[1] Ruđer Bošković Institute, Division of Organic Chemistry and Biochemistry, Bijenička 54, 10000 Zagreb, Croatia; pmance@irb.hr
[2] Ruđer Bošković Institute, Division of Materials Physics, Laboratory for Synthesis and Crystallography of Functional Materials, Bijenička 54, 10000 Zagreb, Croatia; jasminka.popovic@irb.hr
[3] Ruđer Bošković Institute, Division of Physical Chemistry, Laboratory for Chemical and Biological Crystallography, Bijenička 54, 10000 Zagreb, Croatia; zoran.stefanic@irb.hr
[4] Croatian Academy of Sciences and Arts, Nikole Šubića Zrinskog 11, 10000 Zagreb, Croatia
* Correspondence: nsijakov@irb.hr (N.Š.V.); zinic@irb.hr (M.Ž.)

Chemical synthesis of the compounds

General

Reagents were purchased from Aldrich, Fluka, Kemika, Merck and Sigma, and were used without further purification. All solvents were purified and dried according to standard procedures. The reactions were monitored by thin layer chromatography (TLC) on Merck Kieselgel HF254 plastic sheets and spots were made visible using a UV lamp (254 nm) or I_2 vapours. Prepared compounds were purified chromatographically by preparative T.L.C. using silica gel Merck HF254 and by column chromatography using silica 0.063-0.2 mm (Merck).

General procedure for preparation of *N*- alkyl, N′- *L* - methylleucyl(phenylalanyl) oxalamides

Preparation of N-(ethoxyoxalyl)-L-leucine(phenylalanine)methyl ester (**3, 4**); To a cooled (0 °C) solution of hydrochloride of amino acid **1** and **2** (1 mmol) and TEA (2 mmol) in dry CH_2Cl_2 (10 ml) a solution of oxalyl chloride (1 mmol) in dry CH_2Cl_2 (5 ml) was added dropwise during the 15 minutes. The reaction mixture was stirred for 30 min at 0 °C and 20 hours at room temperature. Afterwards, CH_2Cl_2 (10 ml) was added and the mixture was washed successively with 5 % HCl, 5 % $NaHCO_3$ and water. The organic layer was dried over Na_2SO_4 and the solvent was removed under reduced pressure. The ester

obtained in this way was used without additional purification. b) To the solution of N-(ethoxyoxalyl)-L-leucine(phenylalanine)methyl ester (**3**, **4**) in dry CH_2Cl_2 alkylamine was added and the reaction mixture was stirred for 3 days at room temperature. The product was purified by crystallisation (CH_2Cl_2-light petroleum) or preparative TLC chromatography (EtOAc-light petroleum, 100:25).

N-butyloxalamido-L-leucine methyl ester (5)

To the solution of *N*-(ethoxyoxalyl)-L-leucine methyl ester **3** (0.270 g, 1.01 mmol) in dry CH_2Cl_2 (10 ml) n-butylamine (0.110 ml, 1.113 mmol) was added and the reaction mixture was stirred for 3 days at room temperature. The product was purified by crystallisation (CH_2Cl_2-light petroleum) and by preparative TLC chromatography (EtOAc-light petroleum, 200:50). Yield: 78.7 %; $[\alpha]_D$ = - 13 (c 1, CH_2Cl_2); 1H NMR (DMSO-d_6): δ 8.94 (1 H, d, J = 8.3, NH_{Leu}), 8.77 (1 H, t, J = 5.7, NH_{butyl}), 4.36 (1 H, ddd, J=3.6, J=8.3, J=11.1, $CR_{\alpha, Leu}$), 3.63 (3 H, s, OCH_3), 3.18 - 3.09 (2 H, m, CH2N), 1.86 - 1.78, 1.59 - 1.49, 1.48 - 1.39, 1.31 - 1.19 (1H, 2H, 2H, 2H, 4 m, $CH_{\beta, Leu}$, $CH_{2,(\beta, Leu),}$ $CH_{2, butyl}$), 0.89 - 0.83 (9 H, $CH_{3(\gamma, Leu)}$ and $CH_{3, butyl}$), 13 C NMR (DMSO-d_6): δ 171.9 (COOMe), 160.3, 159.4 (CON), 52.0 (OCH_3), 50.4 ($CR_{\alpha, Leu}$), 38.9, 38.5 ($CH_{2(\beta, Leu)}$, $CH_{2Nbutyl}$), 30.7, 19.5 ($CH_{2, butyl}$), 24.3, 22.8, 21.0 ($CH_{\gamma, Leu}$), $CH_{3, \sigma, Leu}$), 13.5 $CH_{3, butyl}$); IR: 3300, 1749, 1652, 1521.

N-hexyloxalamido-L-leucine methyl ester (6)

To the solution of *N*-(ethoxyoxalyl)-*L*-leucine methyl ester (**3**) (1.066 g, 4.346 mmol) in dry CH_2Cl_2 (40 ml) n-hexylamine (0.64 ml, 4.968 mmol) was added and the reaction mixture was stirred for 3 days at room temperature. The product was purified by crystallisation (CH_2Cl_2-light petroleum) and by preparative TLC chromatography (EtOAc-light petroleum, 200:50). Yield: 70 %; [α]D = - 12 (c 1, CH_2Cl_2); 1H NMR (DMSO-d_6): δ 8.93 (1 H, d, J = 8.4, NH_{Leu}), 8.76 (1 H, t, J = 5.8, NH_{hexyl}), 4.36 (1H, ddd , J = 3.5, J = 8.4, J = 10.6, $CH_{\alpha, Leu}$), 3.63 (3 H, s, OCH_3), , 3.16 - 3.08 (2H, m, CH_2N), 1.86 - 1.77, 1.59 - 1.50, 1.49 - 1.41, 1.31-1.18 (1 H, 2 H, 2 H, and 4 H, 4 m, $CH_{\beta, Leu}$, $CH_{2\ (\beta, Leu)}$, $CH_{2,\ hexyyl}$), 0.88, 0.84 (6 H, 2 d, J = 5.9, $CH_{3(\gamma, Leu)}$), 0.86 (3 H, t, J = 6.9, $CH_{3, hexyl}$), 13 C NMR (DMSO-d_6): δ 172.4 (COOMe), 160.8, 159.8 (CON), 52.5 (OCH_3), 50.9 ($CH_{\alpha, Leu}$), 39.4, 39.3 ($CH_{2\ (\beta, Leu)}$, CH_2N), 31.4, 29.1, 26.4, 22.5

($CH_{2, hexyl}$), 24.7, 23.3, 21.5 ($CH_{\gamma, Leu}$), $CH_{3, \sigma, Leu}$), 14.3 $CH_{3, hexyl}$); IR: 3294, 1745, 1687, 1653, 1525.

N-hexyloxalamido-*L*-phenylalanine methyl ester (7)

To the solution of *N*-(ethoxyoxalyl)-*L*-phenylalanine methyl ester (**4**) (4.900 g, 17.544. mmol) in dry CH_2Cl_2 (60 ml) n-hexylamine (2.32 ml, 17.562 mmol) was added and the reaction mixture was stirred for 3 days at room temperature. The product was purified by preparative TLC chromatography (EtOAc-light petroleum, 200:50). Yield: 75 %; 1H NMR (DMSO-d_6): δ 8.92 (1 H, d, J = 8.5, NHPhe), 8.71 (1 H, t, J = 6.0, NH_{hexyl}), 7.32-7.10 (5 H, m, CH_{arom}), 4.57 (1H, dt , J = 5.9, J = 8.5, $CH_{\alpha, Phe}$), 3.64 (3 H, s, OCH_3), 3.17 - 3.01 (4 H, m, CH_2N and $CH_{2(\beta, Phe)}$), 149 - 1.32, 1.32 - 1. 08 (2 H and 4 H, 2m, $CH_{2, hexyl}$), 0.85 (3 H, t, J = 6.8, $CH_{3,hexyl}$), 13 C NMR ($CDCl_3$): δ 170.7 (COOMe), 159.6, 159.0 (CON), 136.4 (C_{arom}), 129.2, 128.8, 127.3 (CH_{arom}), 53.7, 52.5 ($CH_{\alpha, Phe}$ and OCH_3), 39.7, 37.8 ($CH_{2 (\beta, Phe}$, and CH_2N), 31.4, 29.1, 26.5, 22.5 ($CH_{2, hexyl}$), 14.0 $CH_{3, hexyl}$)·

N-dodecyloxalamido-*L*-leucine methyl ester (8)

To the solution of *N*-(ethoxyoxalyl)-L-leucine methyl ester (**3**) (0.372 ml, 1.517 mmol) in dry CH2Cl2 (20 ml), n-dodecylamine (0.35 ml, 1.522 mmol) was added and the reaction mixture was stirred for 3 days at room temperature. The product was purified by crystallisation (CH_2Cl_2-light petroleum) and by preparative TLC chromatography (EtOAc-light petroleum, 200:50). Yield: 70.3 %; $[\alpha]_D$ = - 9.5 (c 1, CH_2Cl_2); 1H NMR (DMSO-d_6): δ 8.93 (1 H, d, J = 8.1, NH_{Leu}), 8.76 (1 H, t, J = 5.6, $NH_{dodecyl}$), 4.39 - 4.32 (1H, m, $CH_{\alpha, Leu}$), 3.63 (3 H, s, OCH_3), 3.12 (2 H, q, J = 6.6, CH_2N), 1.89 - 1.75, 1.61 - 1.50, 1.49 - 1.39 (1 H, 2 H, 2 H, 18 H, 4 m, $CH_{\gamma, Leu}$, $CH_{2 (\beta, Leu)}$, $CH_{2, dodecyl}$), 0.90 - 0.82 (9 H, m, $CH_{3(\gamma, Leu)}$ and $CH_{3, dodecyl}$); 13 C NMR (DMSO-d_6): δ 172.4 (COOMe), 160.8, 159.8 (CON), 52.5 (OCH_3), 50.9 ($CH_{\alpha, Leu}$), 39.4, 39.3 ($CH_{2 (\beta, Leu)}$, $CH_2N_{dodecyl}$), 31.8, 29.51, 29.47, 29.45, 29.42, 29.17, 29.14, 29.10, 26.8, 22.5 ($CH_{2, dodecyl}$), 24.8, 23.3, 21.5 ($CH_{\gamma, Leu}$), $CH_{3, \sigma, Leu}$), 14.4 $CH_{3, dodecyl}$); IR: 3300, 1750, 1656, 1524.

General procedure for preparation of *N* - alkyl, *N'* - *L* – aminoacid amide - oxalamides

The solution of ester in CH_2Cl_2 and conc. NH_3/MeOH was kept for 7 days at 4-8 °C. The precipitate was filtered off, washed with MeOH and dried under reduced pressure or the solvent was evaporated at reduced pressure.

_N_-Butyloxalamido-_L_-leucylamide (9)

The solution of *N*-butyloxalamido-*L*-leucine methyl ester (**5**) (0.380 g, 1.395 mmol) in CH_2Cl_2 (1 ml) and conc. NH_3/MeOH (35 ml) was kept for 7 days at 4 -8 °C. The precipitate was filtered off, and washed with MeOH. Yield: 89 %; 1H NMR (DMSO-d_6): δ 8.78 (1 H, t, J = 6.0, NH_{butyl}), 8.36 (1 H, d, J = 9.0, NH_{Leu}), 7.48, 7.11 (2 x 1 H, 2 s, $CONH_2$), 4.28 (1H, dt, J = 9.3, J = 4.0, $CH_{\alpha, Leu}$), 3.21 - 3.04 (2 H, m, CH_2N), 1.72 - 1.14 (7 H, m, CH_α, $CH_{2(\beta, Leu)}$, $CH_{2, butyl}$), 0.96 - 0.71 (9 H, m, $CH_{3(\gamma, Leu)}$ and $CH_{3,butyl}$); 13 C NMR (DMSO-d_6): δ 173.6 ($CONH_2$), 160.04, 160.01 (CON), 51.8 (CH_α), 41.4, 39.0 ($CH_{2(\beta, Leu)}$, $CH_{2, butyl}$), 31.2, 20.0 ($CH_{2, butyl}$), 24.8 ($CH_{\gamma, Leu}$), 23.5, 21.9 ($CH_{3(\delta, Leu)}$), 14.1 ($CH_{2, butyl}$); IR: 3437, 3385, 3288, 3207, 1687, 1652, 1615, 1519.

_N_-hexyloxalamido-_L_-leucylamide (10)

The solution of *N*-hexyloxalamido-*L*-leucine methyl ester (**6**) (0.375 g, 1.248 mmol) in CH_2Cl_2 (1 ml) and conc. NH_3/MeOH (30 ml) was kept for 7 days at 4 -8 °C. The precipitate was filtered off, and washed with MeOH. Yield: 86.2 %; 1 H NMR (DMSO-d_6): δ 8.78 (1 H, t, J = 6.0, NH_{hexyl}), 8.37 (1 H, d, J = 9.0, NH_{Leu}), 7.48, 7.11 (2 x 1 H, 2 s, $CONH_2$), 4.28 (1H, dt, J = 9.2, J = 3.9, $CH_{\alpha, Leu}$), 3.17 - 3.07 (2 H, m, CH_2N), 1.72 - 1.14 (11 H, m, $CH\alpha$, $CH_{2(\beta, Leu)}$, $CH_{2, hexyl}$), 0.92 - 0.80 (9 H, m, $CH_{3(\gamma, Leu)}$ and $CH_{3,hexyl}$); 13 C NMR (DMSO-d_6): δ 173.6 ($CONH_2$), 160.05, 159.99 (CON), 51.8 ($CH_{\alpha,Leu}$), 41.4, 39.4 ($CH_{2(\beta, Leu)}$, $CH_{2, hexyl}$), 31.4, 29.1, 26.4, 22,5 ($CH_{2, hexyl}$), 24.8 ($CH_{\gamma, Leu}$), 23.5, 21.9 ($CH_{3(\delta, Leu)}$), 14.3 ($CH_{2, hexyl}$); IR: 3432, 3385, 3287, 3208, 1685, 1652, 1515.

_N_-hexyloxalamido-_L_-phenylalaninamide (11)

The solution of *N*-hexyloxalamido-*L*-phenylalanine methyl ester (**7**) (0.023 g, 0.0658 mmol) in CH_2Cl_2 (0.5 ml) and conc. NH_3/MeOH (5 ml) was kept for 7 days at 4 -8 °C. The precipitate was filtered off, and washed with MeOH. Yield: 80.9 %;1H NMR (DMSO-d_6):

δ 8.67 8.67 (1 H, t, J = 5.9, NH_{hexyl}), 8.37 (1 H, d, J = 8.7, NH_{Phe}), 7.58, 7.25 (2H, 2 s, $CONH_2$), 7. 28-7.13 (5 H, m, CH_{arom}), 4.47 (1H, dt , J = 4.5, J = 8.9, $CH_{\alpha, Phe}$), 3.13 - 3.03 (4 H, m, CH_2N and CH_2A $_{(\beta, Phe)}$), 2.99 (1H. dd, J = 9.2, J = 13.7, CH_2B $_{(\beta, Phe)}$), 146 - 1.37, 1.29 - 1. 15 (2 H and 6 H, 2m, $CH_{2\ hexyyl}$), 0.85 (3 H, t, J = 6.9, CH_3, $_{hexyl}$), 13 C NMR (DMSO-d_6): δ 171.9 ($CONH_2$), 159.4, 159.3 (CON), 137.5 (C_{arom}), 129.1, 128.0, 126.3 (CH_{arom}), 54.0 ($CH_{\alpha, Phe}$), 38.8, 35.2 (CH_2 $_{(\beta, Phe)}$ and CH_2N), 30.8, 28.6, 25.9, 22.0 ($CH_{2, hexyl}$), 13.8 CH_3, $_{hexyl}$).

***N*-dodecyloxalamido-*L*-leucylamide (12)**

The solution of *N*-dodecyloxalamido-*L*-leucine methyl ester (**8**) (0.375 g, 0.975 mmol) in CH_2Cl_2 (1 ml) and conc. NH_3/MeOH (25 ml) was kept for 7 days at 4 -8 °C. The precipitate was filtered off, and washed with MeOH. Yield 81.3%;1 H NMR (DMSO-d_6): 8 8.78 (1 H, t, J = 5.9, $NH_{dodecyl}$), 8.36 (1 H, d, J = 9.1, NH_{Leu}), 7.49, 7.11 (2 x 1 H, 2 s, $CONH_2$), 4.28 (1H, dt, J = 9.1, J = 3.8, $CH_{\alpha, Leu}$), 3.18 - 3.02 (2 H, m, CH_2N), 1.72 - 1.09 (23 H, m, CH_α, $CH_{2(\beta, Leu)}$, $CH_{2, decyl}$), 0.91 - 0.81 (9 H, m, $CH_{3(\gamma, Leu)}$ and $CH_{3,dodecyl}$); 13 C NMR (DMSO-d_6): δ 173.6 ($CONH_2$), 160.03, 159.99 (CON), 51.8 (CH_α), 41.4, 39.4 ($CH_{2(\beta, Leu)}$, $CH_{2, dodecyl}$), 31.7, 29.50, 29.47, 29.45, 29.42, 29.17, 29.14, 29.11, 26.8, 22,6 ($CH_{2, decyl}$), 24.8 ($CH_{\gamma, Leu}$), 23.4, 22.0 ($CH_{3(\delta, Leu)}$), 14.4 ($CH_{2, dodecyl}$); IR: 3389, 3291, 3198, 1686, 1653, 1511.

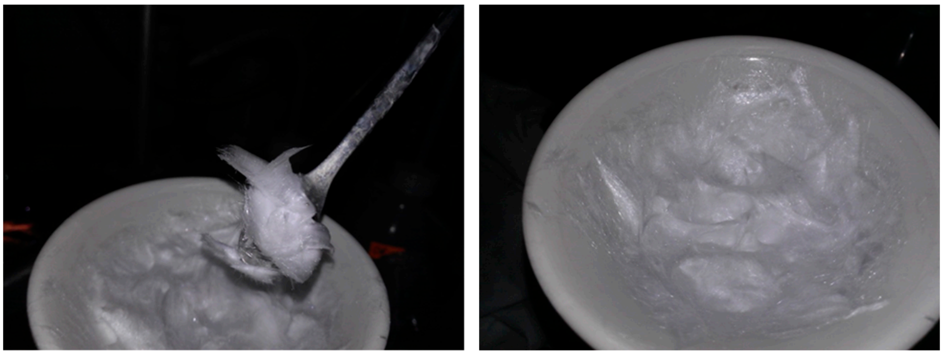

a) **b)**

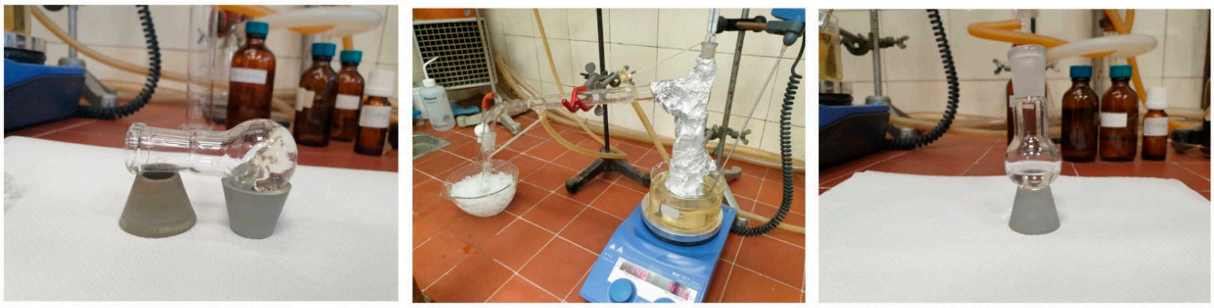

(i) (ii) (iii)

(c)

Figure S1. (a), (b) compound **10** isolated in high purity from methanol solution as the shining cotton-wool like material; (c) Recovery of petrol by distillation of **10** petrol gel.

SEM investigation

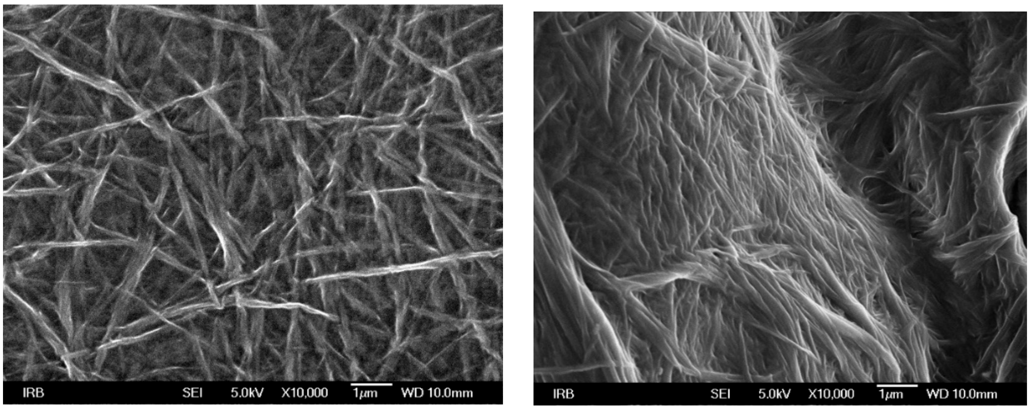

a) b)

Figure S2. SEM images of **10** (a) toluene gel and (b) petrol gel.

DSC study

Table S1. ΔH_m, T_m and T_c values obtained from DSC measurements of petrol, diesel and decaline gel of compound **10** in the heating and cooling cycle.

sample	T_m (°C)	ΔH_m (kJmol^{-1})	T_c (°C)
petrol	129.1	67.9	100.1

diesel	154.3	47.2	128.3
decalin	148.6	41.1	125.2

Single crystal analysis

Crystals of **10** suitable for single-crystal X-ray analysis were grown from methanol by slow evaporation at room temperature. The data collection was performed on an Xcalibur Nova X-ray diffractometer with multilayer optics and Cu Kα radiation (λ = 1.5412 Å) at room temperature.

Table S2. Crystallographic data, structure solution, and refinement for **10** (methanol).

Compound	**10**
Empirical formula	$C_{14} H_{27} N_3 O_3$
Formula weight	285.38
Temperature (° K)	293(2)
Wavelength (Å)	1.542
Crystal system	monoclinic
Space group	$P\ 2_1$
Unit cell dimensions (Å, °)	a = 7.7909(13)
	b = 5.1428(7)
	c = 20.951(5)
	β = 94.502(18)
Volume (Å^3)	836.9(3)
Z	2
Density (calculated) (g cm^{-3})	1.133
Absorption coefficient (mm^{-1})	0.647
F (000)	312
Crystal size (mm)	0.1 x 0.1 x 0.3 mm
Theta range for data collection (°)	4.233-76.026
Index ranges	-6<=h<=9, -6<=k<=6, -25<=l<=26
Reflections collected	4191 / 2590 [R_{int} = 0.0194]
Completeness	99.6 %
Refinement method	Full-matrix least-squares on F^2
Data / restraints / parameters	2590 / 2 / 181
Goodness-of-fit on F^2	1.051
Final R indices [I >2σ(I)]	R_1 = 0.0613, wR_2= 0.1787
R indices (all data)	R_1 = 0.0756, wR_2= 0.2047
Largest diff. peak and hole (e Å^{-3})	0.257 and -0.219

Table S3. Hydrogen bonds for **10** [Å and °].

D-H...A	d(D-H)	d(H...A)	d(D...A)	<(DHA)
a N(1)-H(1)...O(1)#1	0.8600	2.1500	2.948(6)	154.00
b N(2)-H(2)...O(2)#2	0.8600	2.1400	2.958(5)	158.00
c N(1)-H(1)...O(2)	0.8600	2.2900	2.674(6)	108.00
d N(2)-H(2)...O(1)	0.8600	2.3800	2.735(5)	105.00
e N(3)-H(3A)...O(3)#3	0.8600	2.1500	2.998(5)	169.00
f N(3)-H(3B)...O(3)#1	0.8600	2.1800	2.912(4)	143.00

Symmetry transformations used to generate equivalent atoms:
#1 x,y+1,z #2 x,y-1,z #3 -x+2,y+1/2,-z

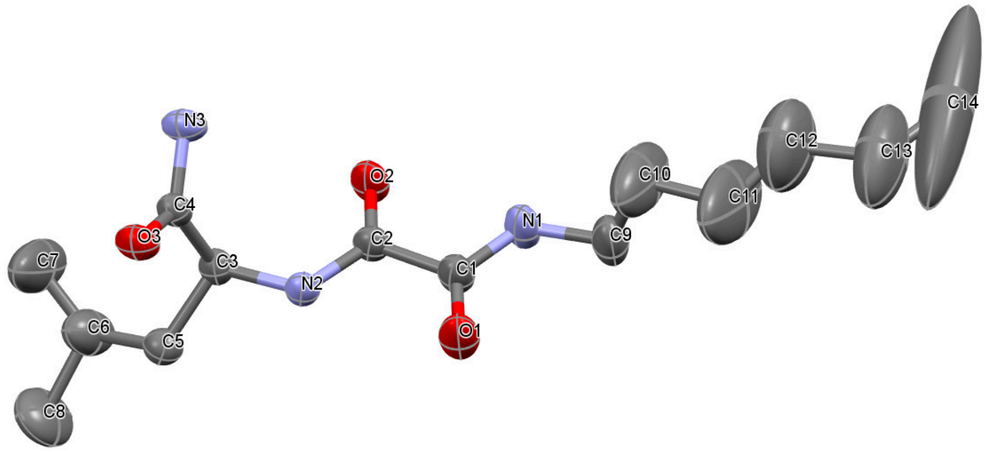

Figure S3. Molecular structure of **10** with the atomic numbering. Displacement ellipsoids are drawn at the 50 % probability level. It is visible that the alkyl chain C9-C14 is highly disordered.

XRPD studies

X-ray powder diffraction data were collected on Philips MPD 1820 with CuK_{alpha} radiation in the range 2.7-20.0 ° 2*θ*, with a step of 0.2° and a fixed counting time of 1 second per step. Additionally, the xerogel sample was grinded in mortar for 3 hours (in order to avoid possible preferred orientation) and measured in the range 2*θ* 2.7- 40.0° with a counting time of 10 seconds per step with the purpose of the space group determination and structure solution.

Structure solution from XRPD data

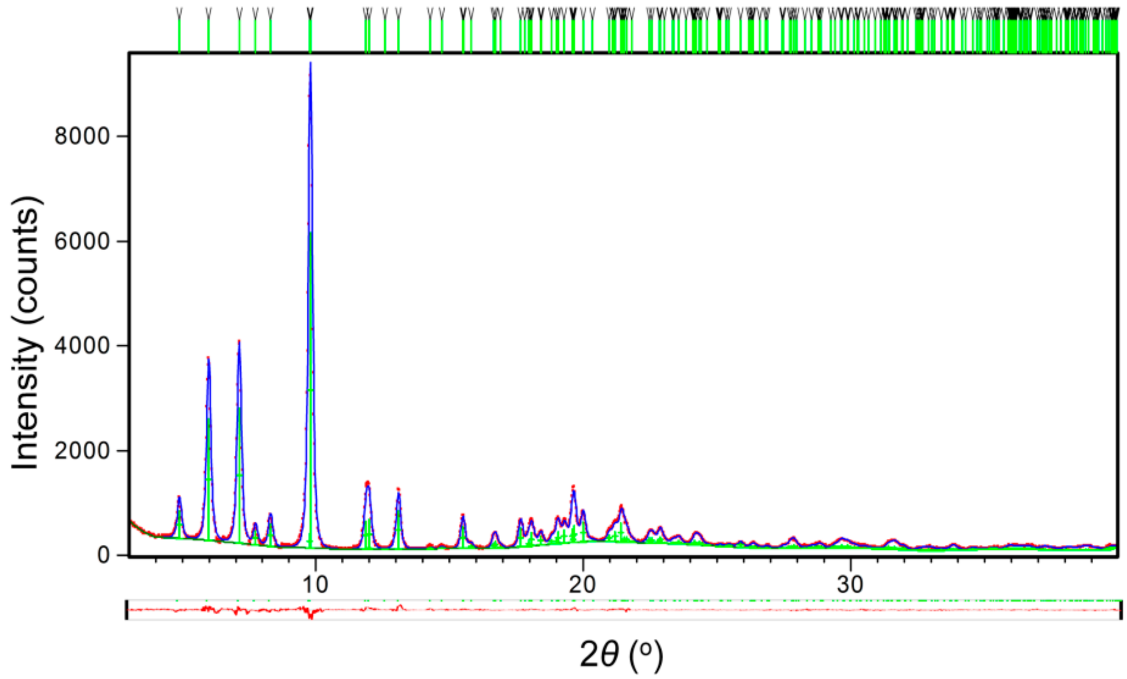

Figure S4. Rietveld refinements on compound **10** in the form of xerogel from toluene. Experimental data are shown as red dots, a calculated profile is shown in blue while the difference curve is provided below. Green vertical marks represent diffraction line positions of compound **10** in the form of xerogel.

Crystallographic data for the structure of **10** (determined from xerogel) are deposited in the Cambridge Structural Database under the CCDC 1524532.

Table S4. Crystallographic data, structure solution, and refinement for **10** (toluene xerogel).

Compound	**10**
Empirical formula	C_{14} H_{27} N_3 O_3
Formula weight	285.38
Temperature (° K)	293(2)
Wavelength (Å)	1.5406
Crystal system	orthorhombic
Space group	*P* 2_1 2_1 2_1

Unit cell dimensions (Å, °)	*a* = 22.837(1)
	b = 29.517(3)
	c = 5.0981(4)
Volume ($Å^3$)	3436.4(5)
F(000)	624
Structure solution	Simulated annealing
Refinement	Rietveld refinement
Theta range for data collection (°)	5-40
R (profile)/ %	6.709
R (weighted profile)/ %	8.739
GOF	2.74

Table S5. Hydrogen bonds for **10** toleune xerogel [Å and °].

D-H···A	*d*(D-H)	*d*(H···A)	*d*(D···A)	<(DHA)
N(1)-H(1)···O(1)	0.8600	2.144	2.936(9)	152.6
N(2)-H(2)···O(2)	0.8600	2.139	2.940(8)	154.7
N(3)-H(3B)···O(3)	0.8600	2.128	2.904(7)	150.5

NMR study

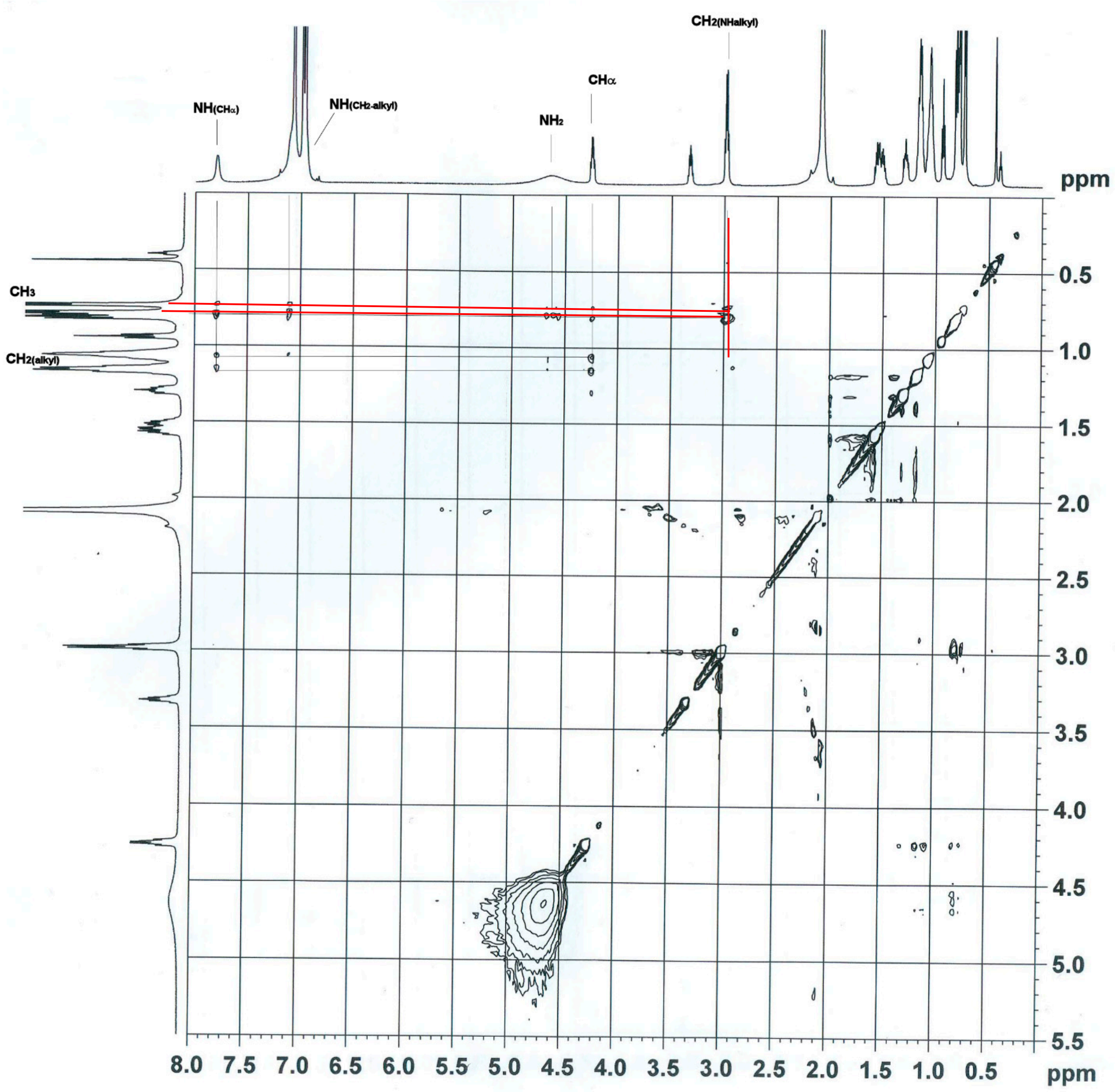

Figure S5. NOSY spectrum of **10** toluene-d8 gel taken at 65°C with indicated intramolecular (black lines) and intermolecular (red lines) *N*-hexyl *α*-methylene (δ 3.0 ppm) / Leu methyl (δ 0.8 ppm) interactions.

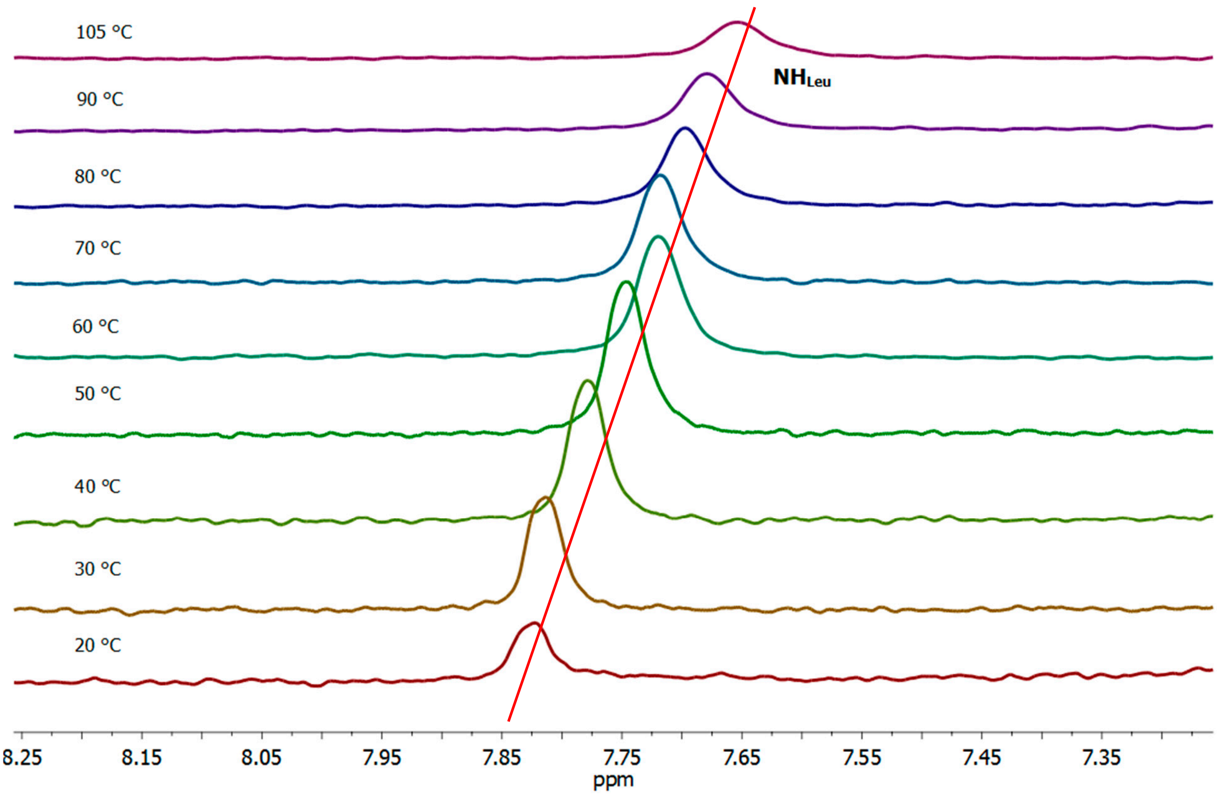

Figure S6. Downfield shifts of the oxalamide LeuNH protons by the temperature variation from 20 to 105°C in the NMR spectra of **10** toluene–d_8 gel. The slope of the red line indicates the extent of temperature-induced downfield shifts for LeuNH protons. The hexylNH protons are positioned under signals of toluene–d_8.

FTIR measurements

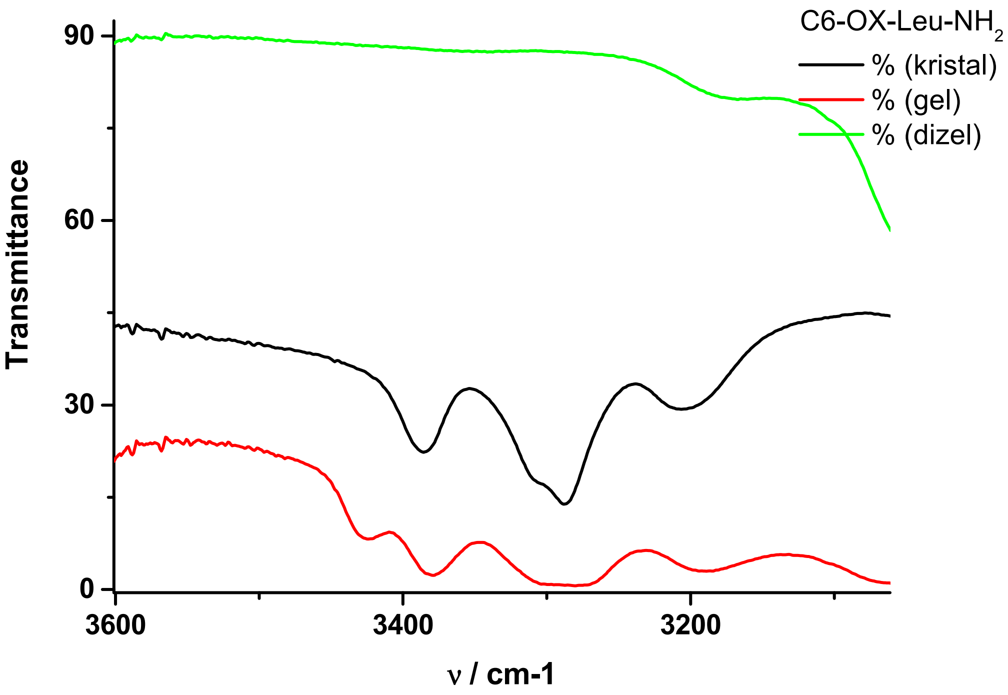

Figure S7. FTIR measurements of **10** crystal (black), diesel gel (red) and diesel solvent (green) at 25°C.
